# Does partial expander deflation exacerbate the adverse effects of radiotherapy in two-stage breast reconstruction?

**DOI:** 10.1186/1477-7819-10-44

**Published:** 2012-02-20

**Authors:** Burcu Celet Ozden, Erdem Guven, Isik Aslay, Gonul Kemikler, Vakur Olgac, Merva Soluk Tekkesin, Bengul Serarslan, Burcak Tumerdem Ulug, Aylin Bilgin Karabulut, Atilla Arinci, Ufuk Emekli

**Affiliations:** 1 Istanbul University, Istanbul Faculty of Medicine, Department of Plastic, Reconstructive and Aesthetic Surgery, Istanbul, Turkey; 2 Istanbul University, Istanbul Faculty of Medicine, Oncology Institute, Department of Radiation Oncology, Istanbul, Turkey; 3 Istanbul University, Istanbul Faculty of Medicine, Oncology Institute, Department of Medical Radiophysics, Istanbul, Turkey; 4 Istanbul University, Istanbul Faculty of Medicine, Oncology Institute, Department of Tumour Pathology, Istanbul, Turkey; 5 Maltepe University, Faculty of Medicine, Department of Plastic, Reconstructive and Aesthetic Surgery, Istanbul, Turkey

**Keywords:** Two-stage breast reconstruction, Radiotherapy, Tissue expansion

## Abstract

**Background:**

The optimum protocol for expander volume adjustment with respect to the timing and application of radiotherapy remains controversial.

**Methods:**

Eighteen New Zealand rabbits were divided into three groups. Metallic port integrated anatomic breast expanders of 250 cc were implanted on the back of each animal and controlled expansion was performed. Group I underwent radiotherapy with full expanders while in Group II, expanders were partially deflated immediately prior to radiotherapy. Control group did not receive radiotherapy.

The changes in blood flow at different volume adjustments were investigated in Group II by laser Doppler flowmetry. Variations in the histopathologic properties of the irradiated tissues including the skin, capsule and the pocket floor, were compared in the biopsy specimens taken from different locations in each group.

**Results:**

A significant increase in skin blood flow was detected in Group II with partial expander deflation. Overall, histopathologic exam revealed aggravated findings of chronic radiodermatitis (epidermal atrophy, dermal inflammation and fibrosis, neovascularisation and vascular changes as well as increased capsule thickness) especially around the lower expander pole, in Group II.

**Conclusions:**

Expander deflation immediately prior to radiotherapy, may augment the adverse effects, especially in the lower expander pole, possibly via enhanced radiosensitization due to a relative increase in the blood flow and tissue oxygenation.

## Background

Tissue expander assisted two-stage breast reconstruction has been successfully utilized in many breast centers throughout the world [1,2]. Furthermore, favorable results have recently been reported with the use of this approach in patients with locally advanced breast cancer requiring postmastectomy radiotherapy [3], leading to an increase in the number of patients in whom anatomical breast expanders are inserted at the time of mastectomy. However, the optimal protocol for expander volume adjustment with respect to the timing and application of radiotherapy (RT) remains controversial. While deflation of the expander at the time of RT helps to create a favourable geometry for dose planning, attempts to re-inflate after radiotherapy may cause extrusion through skin, complicating the reconstruction process [4,5]. On the other hand, there are reports of successful outcomes with the application of RT whilst the expanders remained fully inflated [1,6,7].

In order to compare the effects of RT on pre-expanded tissues with different expander volume adjustments, we designed an experimental study with anatomically shaped breast expander implanted rabbits, in which RT was applied to pre-expanded tissues with expanders in an either fully inflated or partially deflated state. The changes in blood flow at different volume adjustments and the variations in the histopathological properties of the irradiated tissues including the skin, capsule and the pocket floor, were investigated.

## Methods

This study was performed at the Institute for Experimental and Applied Medical Research of Istanbul University with permission of the Ethics Committee of the Istanbul University, Istanbul Faculty of Medicine.

Eighteen female New Zealand white rabbits (3.0-3.5 kg) were randomly divided into 3 groups (Table 1). The animals were anesthetized by intramuscular injection of ketamine (35 mg/kg) (Ketalar; Parke-Davis, Eczacibasi; Istanbul, Turkey) and xylazine (5 mg/kg) (Rompun; Bayer Istanbul, Turkey). Anesthesia was maintained by using the same two agents intraperitoneally.

**Table 1:** Expander status at the time of radiotherapy in different groups.

*Groups*	*RT at 6^th ^wk*	*Expander Status (during RT)*	*Sacrification* *(Postoperative wks)*
**I (n = 6)**	Yes	Fully inflated	12 wks
**II (n = 6)**	Yes	Partially deflated	12 wks
**Control (n = 6)**	No	Fully inflated	12 wks

### Experimental Time Table

**Figure 1:**
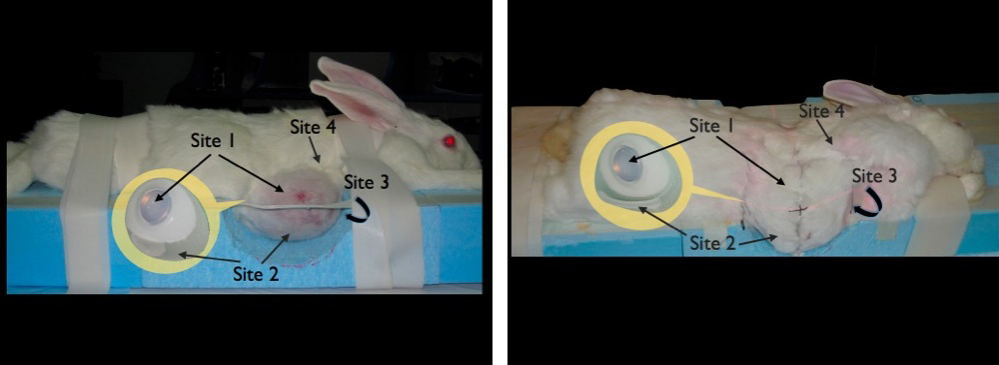
**Expander status at the time of radiotherapy**. Definition of sites that have been investigated. **1a**- Left, Expanders were fully inflated in Group I. **1b- **Right, Expanders were partially deflated in Group II. **Site 1: **The superior portion of the expander, i.e the tissues that lay over the metallic port; including epidermis, dermis and capsule. **Site 2: **The lower/most prominent portion of expander; including epidermis, dermis and capsule. **Site 3: **The floor of the expander pocket, including capsule and connective tissue. **Site 4: **Neighbouring tissues in the non-expanded area adjacent to the expander pocket.

The experiment ran 12 weeks.

a. A 250 cc contour profile breast expander with metallic port (Mentor Corporation, Santa Barbara, Calif.) was placed dorsolaterally on one side of each rabbit in Groups I, II and Control.

b. Starting from the second postoperative week, the expanders were gradually inflated to full volume by instillation of 50 cc saline every four days over a period of three weeks.

c. Radiotherapy was given at the end of the sixth postoperative week in Groups I and II. Control group did not receive radiotherapy.

d. Group I underwent radiotherapy with the expander in a fully inflated state (Figure 1a). The expanders in Group II were deflated to 1/3 of its original volume, 24 hours before radiotherapy (Figure 1b).

e. Re-inflation of the expanders in Group II was started at 2 weeks following radiotherapy and pre-deflation volume was reached with three sessions of saline instillation, performed over a period of two weeks.

f. Laser Doppler flowmetry (LDF) measurements were taken in Group II at different states of expander volume adjustments at the start and after completion of radiotherapy.

g. All animals were sacrificed at the twelfth postoperative week; biopsies were taken for histopathological examination.

### Operative Technique

The surgical field was prepared by electrical clipping followed by chemical depilation to achieve total hair removal. Skin disinfection was achieved by using 10% povidone-iodine, and a single dose of cefazolin (60 mg/kg) was administered intramuscularly as a prophylaxis against infection. Following a 5-cm incision, parallel to the spine, a 250 cc contour profile breast expander with metallic port (Width: 10.1 cm., Height: 10.7 cm., Projection: 5.6 cm., Mentor Corporation, Santa Barbara, Calif.) was placed dorsolaterally, over the 4-12^th ^ribs, on the right side of each rabbit into a surgically created pocket, underneath the panniculus carnosus muscle, away from the incision. Care was taken to create a pocket, which would fully accommodate the expander base without any folds while avoiding excessive dissection, which would lead to expander rotation and/or dislocation. The subcuticular layer adjacent to the upper margin of the expander was fixed to the thoracic wall with several 4/0 polyglactin sutures to confine the expander in the pocket away from the incision. The skin was closed using 4-0 polypropylene sutures.

### Postoperative Care

The animals were held under a 12-hour dark at 22-/+ 2°C and housed separately for twelve weeks. They were observed twice weekly for general condition, weight loss, systemic and operative site infection, seroma formation as well as changes in expander volume and position.

### CT Planning

**Figure 2:**
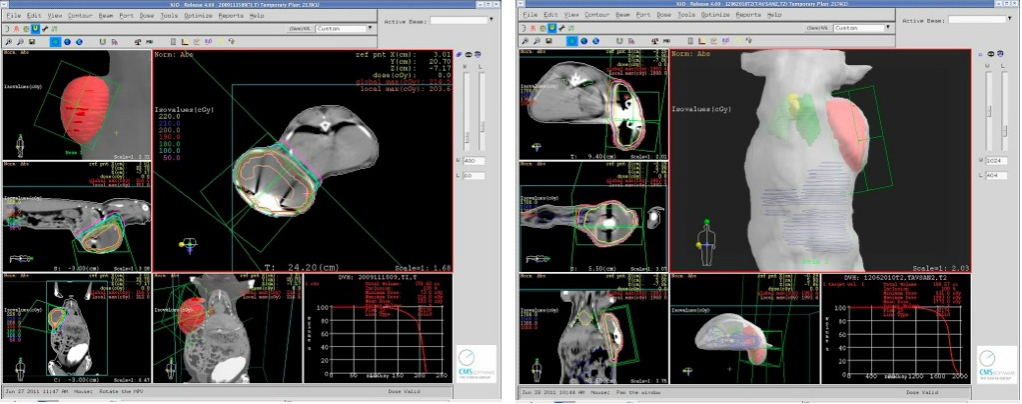
**Treatment planning and isodose lines in the experimental groups I (2a, Left) and II (2b, Right)**.

Customized mold for animals was made of Styrofoam for immobilization during acquisition of the computerized tomography (CT) scans to be used for planning and irradiation. The animals were anesthetized and immobilized in prone position inside the mold. Continuous anesthesia was maintained for each animal during both CT planning and the subsequent delivery of radiotherapy. The fixation was carefully done by a surgical tape before the CT scan in order to minimize changes in positioning during the process of planning and irradiation. Following the immobilization and positioning, all animals underwent CT (Brilliance CT - Big Bore Oncology, Phillips, Holland) scanning in the irradiation position for 3D treatment planning. CT slices were taken at 3-mm intervals and 5-mm slice width. After CT scanning, the entire animal's CT data set was transferred to the *CMS XiO *treatment planning system (Elektra, Sweden). The external contour and prosthesis volume were delineated on the axial CT scans. The target volume to be irradiated was defined by the expander volume (Figures 2a and 2b).

All dose plans were designed for Co-60 gamma beams. Dose distribution was calculated using two opposed tangential fields. The posterior borders of the fields were aligned to minimize dose for healthy tissues. Both the gantry and collimator angles and the field sizes were defined by the planning.

### Irradiation Protocol

Immediately after CT planning, the animals were irradiated with the isocenter at 80 cm at a Cirus cobalt teletherapy unit (Cis Bio International, France). Dose prescription was 17 Gy at single fraction.

### Dynamic Blood Flow Analysis

Laser doppler flowmetry (LDF) analysis was used to investigate dynamic blood flow in various parts of normal and expanded skin (Vasamedics, Laserflo BPM^2^, St. Paul, MN). The output was recorded in mL/min/100 g. LDF recordings were taken in Group II, over three locations at each measurement session; Sites 1, 2 and 4 (Figure 1b). LDF recordings were taken at the end of the sixth postoperative week, with expanders in a fully inflated state, following partial deflation prior to radiotherapy, as well as at the time of completion of the re-inflation process. All recordings were taken after a 24-hour period following final volume adjustment of the expander in order to ensure the stabilization of blood flow according to the new expander pressure. The LDF was zeroed on the neutral colored faceplate and all measurements were done after the probe, heated to 40°C, had been placed flat on shaved skin for 60 seconds. Standard probe adhesive strips were used to keep probe-skin distance equal.

### Histopathologic Examination

The expanded skin and capsule were harvested and stored in formalin for histopathological processing. Four different sites from each specimen groups were selected and evaluated (Figure 1a and 1b).

The specimens were embedded in paraffin, cut in approximately 5 μm thick sections and stained using hematoxylin and eosin (H&E). Immunohistochemical staining was done by CD31 primary antibody.

Following parameters were analyzed in each biopsy site of every animal.

1) Epidermal changes (Presence of ulceration, Epidermal thickness in μm/pixel)

2) Dermal changes (Presence of inflammation, fibrosis and hyalinization of dermal collagen)

3) Vascular changes (Dilatation, hyalinization, proliferative changes in vessel walls)

4) Neovascularisation coefficient (Thin-wall vessels were counted in 10 microscopic high-power fields and averaged out)

5) Capsular Properties (Composition and thickness in mm/pixel)

The specimens were examined in Olympus BX60 microscope attached to a color video camera and connected to a computer. Images were captured using the camera and displayed on the computer monitor. All measurements were done with Olympus Image Analysis System 5.

### Statistical Analysis

NCSS (Number Cruncher Statistical System) 2007&PASS 2008 Statistical Software (Utah, USA) program was used for the statistical analysis. The numerical results of the experimental groups were compared with the Kruskal Wallis test and post hoc analysis was conducted with the Mann Whitney U test. Intragroup comparisons were performed with the Friedman test and Wilcoxon Signed Rank test. Categorical data were evaluated with Fisher's exact test. Probability values less than 0.05 were considered significant.

## Results

The postoperative course was uneventful in each group. No animals were lost due to surgery or radiotherapy. There were no findings of postoperative infection, hematoma or expander extrusion. Seroma formation following radiotherapy was observed in 2/6 animals in Group II, whereas none developed in Groups 1 and Control (p = 0.45). No intervention was required and it resolved spontaneously following re-inflation in both cases. No expanders were lost in any of the groups.

### Dynamic blood flow analysis

**Figure 3:**
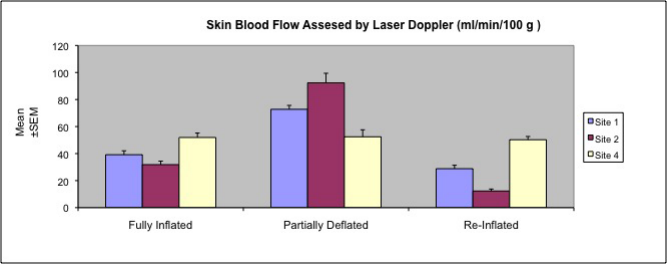
**Skin blood flow at different states of expansion, in Group II**.

A significant increase in blood flow was observed with partial expander deflation. This effect was more prominent in the lower expander pole. Re-inflation following radiotherapy caused a significant drop in the blood flow values, especially in the lower pole (Table 2 and Figure 3).

**Table 2:** Dynamic blood flow analysis in Group II animals at different states of expansion.

Blood Flow (ml/min/100 g)	Start of RT		4 wks. after RT
	
	Fully inflated (FI) Median (Range) *	Partially deflated (PD) Median (Range) *	Re-inflated (RI) Median (Range) *
**Site 1**	39 (29-50)^b^	73 (61-82)^a^	29 (25-36)^b^

**Site 2**	32 (22-40)^b^	87 (76-122)^d^	12 (7-17)^f^

**Site 4**	51 (41-65)^e^	54 (31-66)^e^	49 (42-61)^e^

*Results in the same row (Friedman test) or column (Wilcoxon Signed Ranks test) but with different superscripts differ significantly (p < 0.01 and p < 0.05 respectively)

### Histopathologic findings

#### Epidermal Changes

***Skin ulceration***, mainly in the lower expander pole, was observed in four of the animals in Group II whereas none was evident in Groups 1 and Control (p > 0.05).

**Figure 4:**
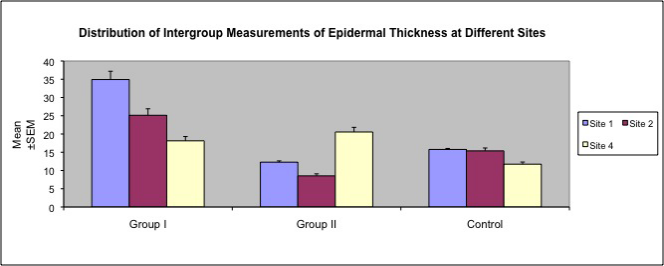
**Distribution of Epidermal Thickness Values**.

**Figure 5:**
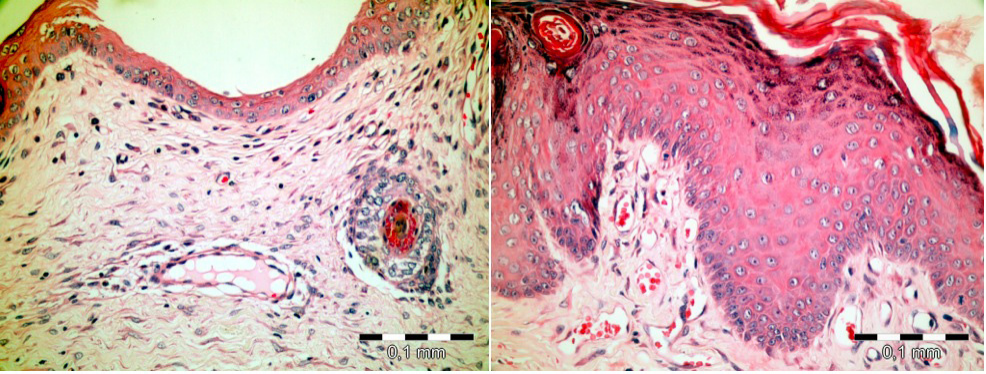
**Epidermal changes in the experimental groups**. Left: Epidermal atrophy in the lower expander pole in Group II. Right: Epidermal hyperplasia over the expander port in Group I (H&E, X400).

***Epidermal thickness ***measurements in those sites overlying the expander were significantly higher in Group I when compared with the Control group *(p *< 0.01). Contrarily, Group II values were significantly lower than the Control group *(p *< 0.01) (Table 3 and Figure 4). Epidermal atrophy in Group II, was more prominent in the lower expander pole, whereas marked epidermal hyperplasia was observed over the expander port in Group I (Figure 5).

**Table 3:** Epidermal thickness measurements at different sites.

Epidermal Thickness (μm/pixel)	Group I	Group II	Control
	
	Median (Range) *	Median (Range) *	Median (Range) *
**Site 1**	33.4 (28.2-43.3)^a^	12.4 (10.8-13.2)^d^	15.8 (14.8-16.7)^f^

**Site 2**	25 (18.7-31.3)^b^	8.8 (6.4-10.2)^e^	15.7 (11.6-17.3)^g^

**Site 4**	19.2 (12.6-20.4)^c^	19.7 (16.7-25.6)^c^	11.1 (10.4-14.2)^h^

*Results in the same row (Kruskal Wallis test) or column (Wilcoxon Signed Ranks test) but with different superscripts differ significantly (p < 0.01 and p < 0.05 respectively)

#### Dermal Changes

##### Dermal Inflammation

**Figure 6:**
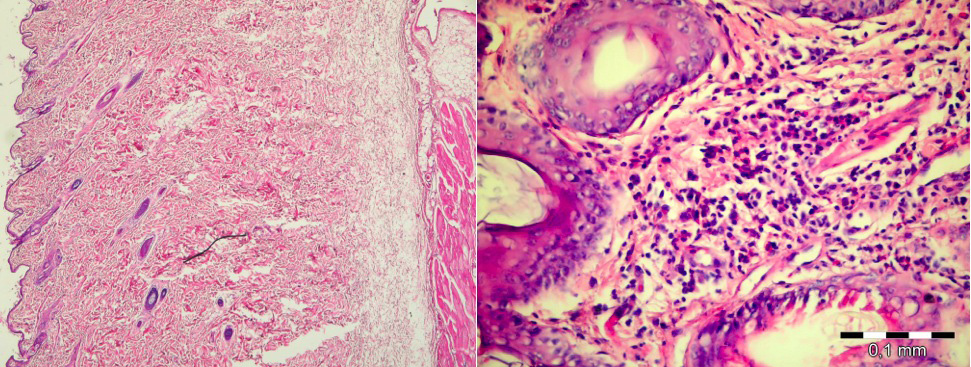
**Dermal changes in the experimental groups**. Left: Dermal inflammatory findings are minimal in the lower expander pole in Group I (H&E, X40). Right: Prominent dermal inflammation in the lower expander pole, in Group II, (H&E, X400).

The presence and intensity of interstitial mononuclear inflammatory infiltrates were found to be increased, especially in the lower pole samples from Group II when compared with Groups I and Control (Figure 6).

##### Dermal Collagen Changes

Similarly, dermal fibrosis and hyalinization of dermal collagen were mainly observed in Group II, when compared with Groups I and Control.

#### Vascular Changes

**Figure 7:**
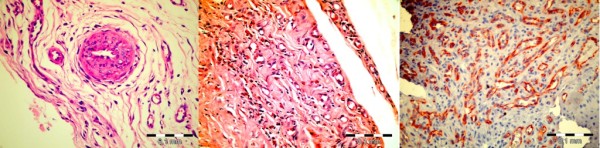
**Vascular changes in the lower expander pole in Group II**. Left: Media proliferation (H&E, X400). Center and Right: Neovascularisation (H&E, X400 and CD 31, X400 respectively).

Presence of vascular dilatation, hyalinization and proliferative changes of the vessel walls were prominent in all three sites in Group II whereas those findings were minimal or absent in Groups I and Control (Figure 7).

##### Neovascularisation

Neovascularisation values of Group II were significantly higher than both Groups I and Control *(p *< 0.01). Group I values were also significantly higher than the control group in all three sites *(p *< 0.01) (Table 4).

**Table 4:** Neovascularisation values at different sites

Neovascularisation (no. of thin vessel walls per high power field)	Group I Median (Range) *	Group II Median (Range) *	Control Median (Range) *
**Site 1**	19 (16-22)^a, A^	28 (24-36)^b, B^	10 (8-13)^c, C^

**Site 2**	16 (14-20)^e, A^	33 (28-36)^d, B^	12 (9-14)^f, C^

**Site 3**	15 (12-19)^h, A^	29 (25-35)^g, B^	11 (9-13)^c, C^

*Results in the same row but with different small case superscripts(Kruskal Wallis test) and results in the same column but with different capital superscripts(Wilcoxon Signed Ranks test) differ significantly. (p < 0.01 and p < 0.05 respectively).

#### Capsule Changes

##### Composition

The capsule reaction was mainly fibrous at all sites in Group II whereas a fibrocellular/cellular quality was predominant in Groups I and Control.

##### Capsule Thickness

**Figure 8:**
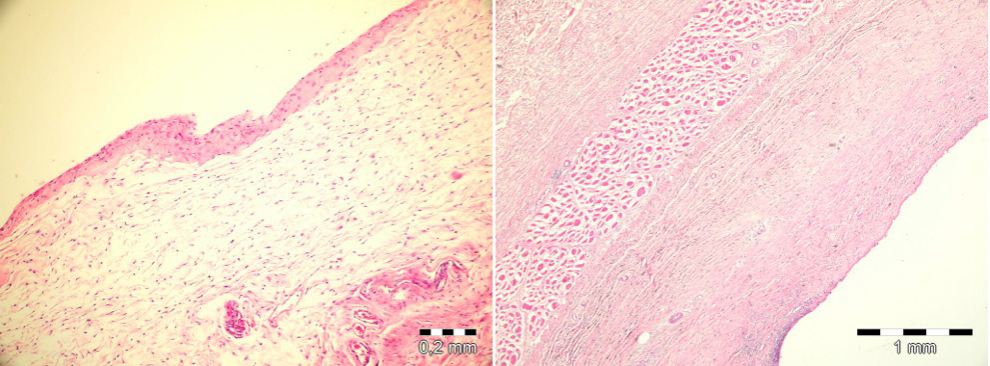
**Capsular changes in the experimental groups**. Left: Thin, fibrocellular capsule in Group I (H&E, X100). Right: Thick, fibrous capsule in Group II (H&E, X40).

Capsule thickness at sites overlying the expander were significantly higher in Groups I and II than Control. *(p *< 0.01 and *p *< 0.01 respectively). Overall capsule thickness was found to be higher in Group II than Group I (Figure 8).

**Figure 9:**
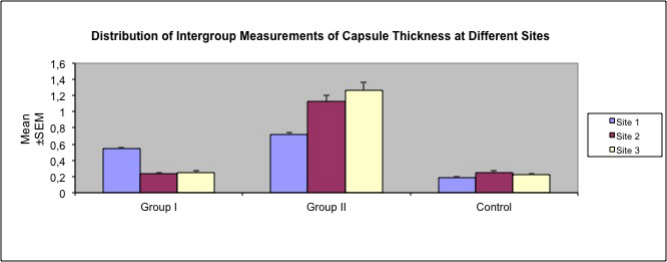
**Site and group specific variations in capsule thickness**.

According to the intragroup comparisons, capsule thickness over the port was found to be significantly higher than the lower pole and the pocket floor in Group I *(p *< 0.05), whereas significantly lower values in the same site were observed in Group II *(p *< 0.05) (Table 5 and Figure 9).

**Table 5:** Capsule thickness measurements at different sites.

Capsule Thickness (mm/pixel)	Group I	Group II	Control
	
	Median (Range) *	Median (Range) *	Median (Range) *
**Site 1**	0.54 (0.48-0.61)^a, A^	0.73(0.6-0.86)^b, C^	0.16 (0.11-0.28)^c, E^

**Site 2**	0.22 (0.17-0.26)^d, B^	1.1 (0.9-1.46)^e, D^	0.24 (0.18-0.35)^d, E^

**Site 3**	0.22 (0.19-0.39)^f, B^	1.25 (0.89-1.71)^g, D ^	0.21 (0.18-0.28)^f, E^

*Results in the same row but with different small case superscripts (Kruskal Wallis test) and results in the same column but with different capital superscripts (Wilcoxon Signed Ranks test) differ significantly (p < 0.01 and p < 0.05 respectively).

## Discussion

Although nipple sparing and skin sparing mastectomy techniques mostly maintain the original dimensions of the breast skin envelope, a variable amount of skin resection is usually needed at the time of mastectomy, depending on the tumor site and the concerns on the marginal skin flap viability. Thus, immediate expander fill volumes usually fail to reach the final desired amount [8], and often several sessions are needed to expand the skin to its original size following mastectomy. Although, the classical effects of tissue expansion may not be observed in those cases in whom the expander serves solely to preserve the native breast skin, dermal and vascular structure changes accompany those cases where skin resection necessitates postoperative expansion to original breast size for optimum cosmesis.

The compatibility of tissue expansion and radiotherapy has been investigated in various studies. The results seem to vary according to the timing of delivery of radiotherapy and the execution of actual tissue expansion. Most of the studies that report on the adverse effects of radiotherapy have either placed the expanders in previously irradiated skin or begun the radiation therapy during the expansion period [9,10]. However, according to an experimental study on rabbits, the effects of radiotherapy may not be as deleterious, when delivered on a pre-expanded field, with an actual increase in epidermal thickness and vascularity with increasing doses of radiotherapy [10]. This has been attributed to the probability of a better tolerance to radiotherapy after a period of tissue expansion and stabilization [10]. Having said this, it has not been investigated how this tolerance will change when radiotherapy is delivered to an expanded-deflated area and if subsequent re-inflation will change the tissue tolerance to the adverse effects of radiotherapy. In our study we looked into the chronic effects of radiotherapy, when delivered on a previously expanded, stabilized and partially deflated area and compared it with the effects of radiotherapy delivered on a fully inflated field. Previous studies on chronic effects of radiotherapy on implant sites, have underlined the presence of epidermal changes (either hyperplasia or atrophy), dermal edema and connective tissue degeneration, neovascularisation and vascular changes (dilatation, vessel wall proliferation, thrombi formation) as well as increase in fibrous capsule thickness [10-12]. Our findings on the chronic effects on radiotherapy when executed at full expansion are compatible with the prior studies [10,11]. However, we have further shown that, expander deflation, immediately prior to the delivery of radiotherapy, frankly exacerbates the adverse radiation effects.

Another point of interest related to the current study is the use of metallic port-integrated, anatomic tissue expanders, to better mimic the clinical situation. What we found with the use of such expanders was a variation in the region specific radiotherapy effects, i.e aggravated findings in the lower pole as compared to the sites neighboring the magnetic port. Previous ex vivo studies have shown that metallic port related dose attenuation is in fact present, albeit minimal and may be either overcome or overlooked in clinical practice [13,14]. Our study findings of comparingly subdued RT effects over the magnet area in the deflated group, may indirectly be considered as concurrent with the aforementioned preliminary data [13,14]. However, a relatively augmented, regional expansion-deflation effect due to anatomic expander configuration, may also have contributed to the aggravated findings in the lower pole.

Laser Doppler flowmetry (LDF), has been found to be a suitable method for the registration of reactions in dermal circulation, caused by tissue expander pressure changes [15]. A statistically significant association between the changes of the LDF values and the intraluminal pressure was observed [15]. The relationship between tissue expansion and cutaneous blood flow has also been studied by Goding et al [16]. Their study is unique due to its investigation of the effects of expander deflation on the pig skin circulation. Briefly, expanders were inserted into subcutaneous pockets in two experimental groups and a separate group with no expanders served as control. Controlled expansion followed by deflation was executed in Group I, while the expanders were kept non-deflated in Group II. According to their results, expander deflation following controlled tissue expansion causes an immediate increase in blood flow. However, this effect is reported to be temporary and furthermore, six days after deflation, skin expansion had a negative impact on cutaneous blood flow as compared to skin elevated without expander placement [16]. In our study, the LDF measurements were compatible with the reported results, confirming a significant early increase in the blood flow by expander deflation. Furthermore, in Group II, attempts for re-inflation two weeks after completion of radiotherapy, which is compatible with the expander re-inflation timing in the clinical setting [3], yielded a significant drop in skin blood perfusion, detected by the third LDF readings. This finding of relative skin ischemia may also help to explain the re-inflation related problems including expander extrusion and loss, reported in large clinical series by Kronowitz et al. [3].

Molecular oxygen is known to be a potent modifier of cellular radiation sensitivity and the biological effects of ionizing radiation on mammalian cells are reported to be aggravated under well- oxygenated conditions [17]. Thus, increased tissue perfusion and better oxygenation in the deflated state, may well account for the underlying etiopathology of the exacerbated adverse effects, observed in our study.

Clinically, when Kronowitz et al. first introduced the concept of delayed- immediate breast reconstruction, complete deflation of the expanders prior to radiotherapy was recommended, favoring a more effective addressing of the internal mammary nodes while avoiding adverse effects [5,18]. Subsequently, due to high rates of expander loss during re-inflation, the protocol was revised and from then on, the expanders were kept partially deflated during the course of radiotherapy [6]. On the other hand, it has been clinically observed that even partial deflation may cause problems; Expander/skin dimpling and formation of a prominent inferior edge is one of the culprits that may lead to skin trauma facilitating exposure. Furthermore, deliberate dimpling of the expanded skin is reported to cause unacceptably high variations in terms of dosimetry [19] and dosimetric discrepancies greater than 10% of the original treatment dose are considered unacceptable in radiotherapy [20]. Since such unwanted variations have been attributed to changes in the size and shape of the underlying tissue expander [19], it may be a reasonable suggestion to stabilize the pocket with a fully inflated, taut implant and avoid fluctuations while executing RT.

In one study by Ascherman et al, it is stated that radiotherapy may be given before completion of expansion, to be continued following completion of radiotherapy [6]. In such cases, an increase in the complication rate was not observed. However, although it may be presumed that interrupted expansion does not cause as strong adverse effects as those under the hypervascular conditions that accompany a deflated expander after full expansion has been reached, cosmetic consequences of irradiating an insufficiently developed skin envelope and trying to expand it to the desired volume following radiotherapy may not be as satisfactory as those achieved with the irradiation of an already established skin envelope [7].

## Conclusions

Expander deflation immediately prior to radiotherapy, increases adverse effects, potentially complicating the reconstruction process. To avoid radiotherapy-related problems, keeping the expanders in the fully inflated state may be an option. In those patients where optimum dose planning cannot be executed with full expanders, approximately one week wait period following partial deflation may be suggested before executing radiotherapy, in order for the hypervascular state in the tissues to be settled back to normal values.

## Competing interests

The authors declare that they have no competing interests.

## Authors' contributions

All of the authors have made substantial contributions to conception and design, and/or acquisition of data or analysis and interpretation of data. BCO conceived of the study, and along with EG participated in its design and coordination. IA, GK and BS planned and carried out the radiotherapy process. VO and MST performed the histopathologic examination. BT, ABK and AA helped interpret the findings and draft the manuscript. All authors read and approved the final manuscript.
